# Supplementary material for: The effect of artificial selection on phenotypic plasticity in maize
Source: Nat Commun. 2017 Nov 7;8:1348. doi: 10.1038/s41467-017-01450-2 (PMC5677005; doi:10.1038/s41467-017-01450-2)
Supplement: Supplementary file 1 — Supplementary Information [file 41467_2017_1450_MOESM1_ESM.pdf]

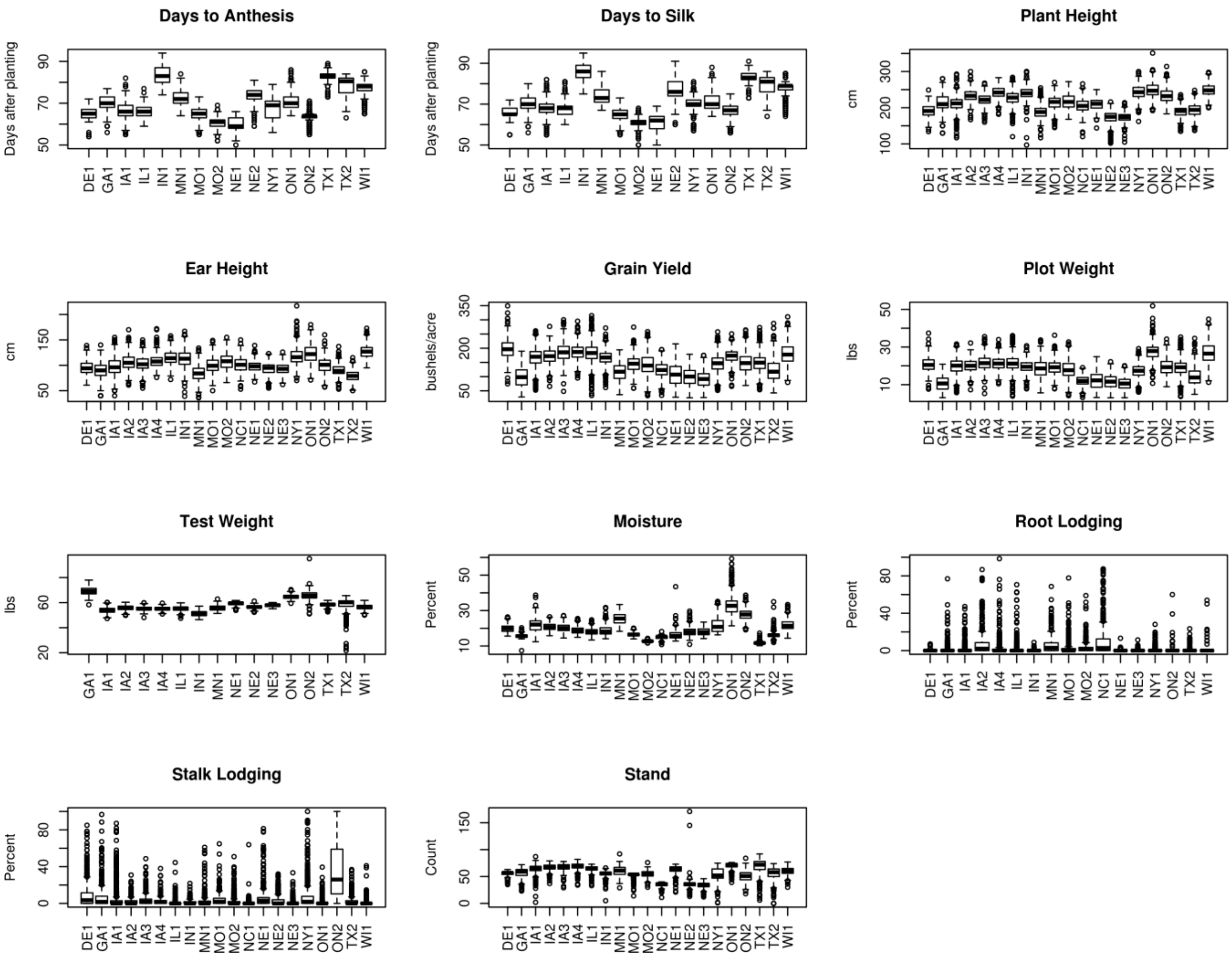

<u>Supplementary Figure 1: Range of observed phenotypes</u>

Phenotypic values for 11 traits measured in up to 21 environments. Whiskers of the boxplots represent 1.5 times the interquartile range of the data, and the middle is marked at the median of the observations. Not all traits were measured in all environments.

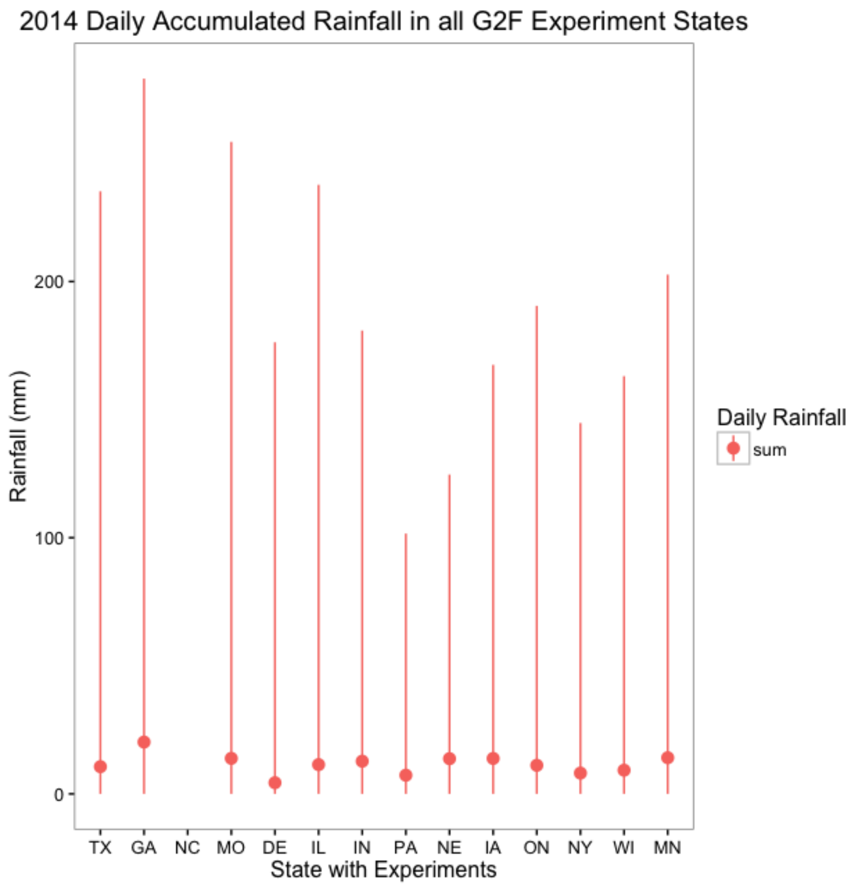

Supplementary Figure 2: Daily precipitation ranges

Range of daily precipitation for each state. Some states represent more than one experimental site. Dots represent mean values, while the bars cover the entire range of observed values. No rainfall data is presented for North Carolina due to equipment malfunctions.

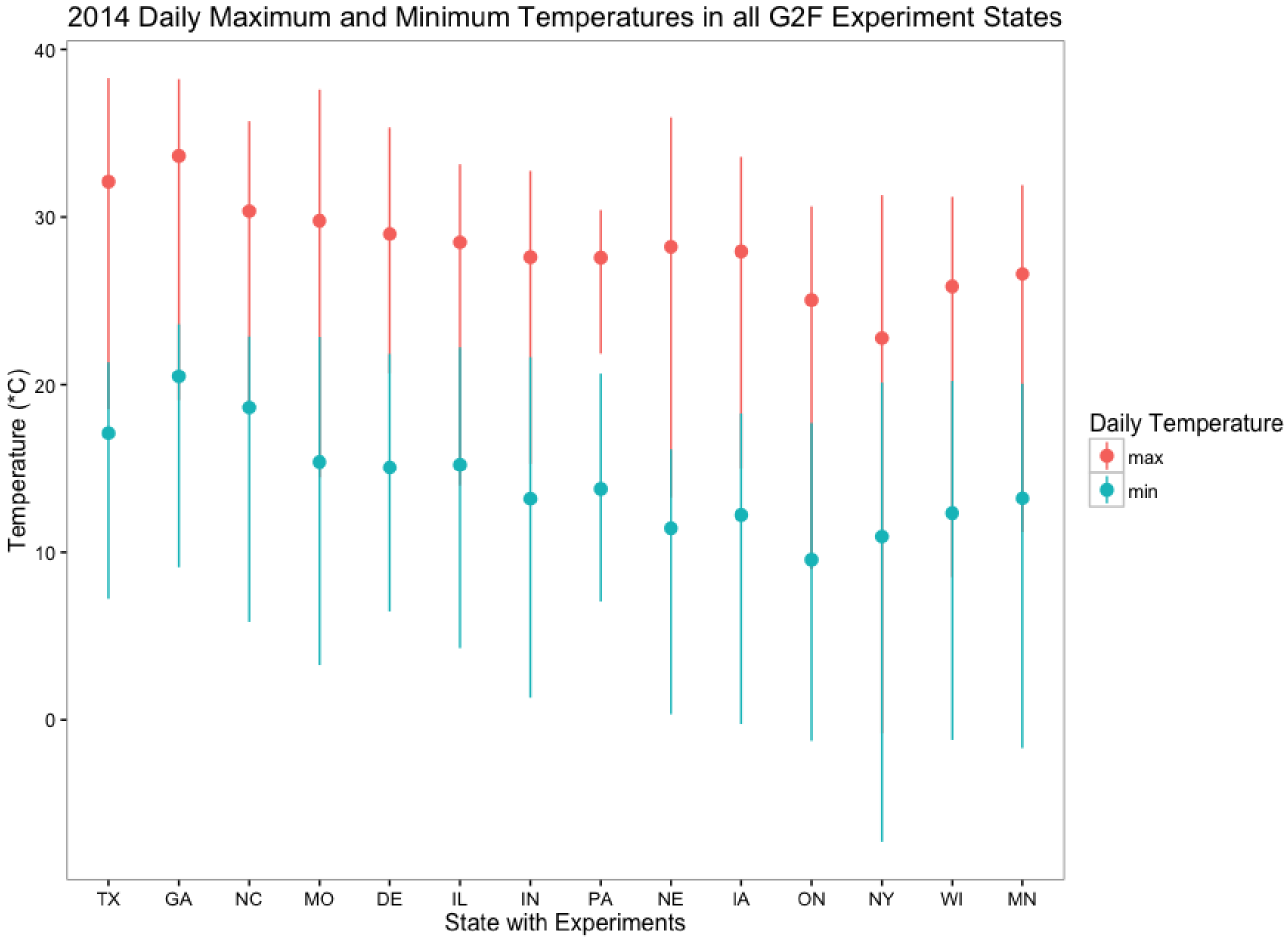

Supplementary Figure 3: Daily temperature ranges

Range of minimum and maximum daily temperature for each state. Some states represent more than one experimental site. Dots represent mean values, while the bars cover the entire range of observed values.

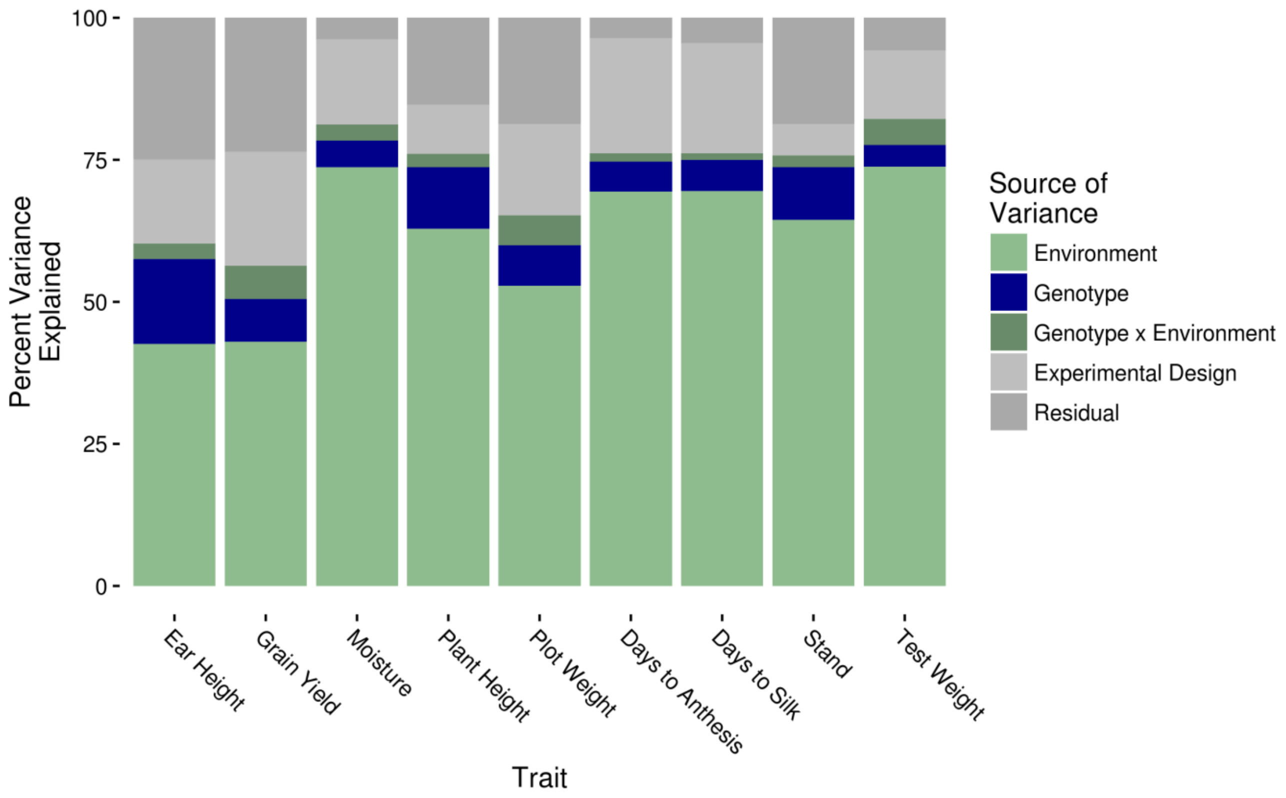

Supplementary Figure 4: Experimental design variance component estimation

Modified split-plot random effects models were fit for each trait, and percent variance attributable to each model term calculated. Genotypic variance is represented by the hybrid line within set term of the model; G×E variance is represented by the hybrid line by environment within set term of the model; experimental design variance is a pool of set, environment by set, replication within environment, and replication by set within environment terms of the model.

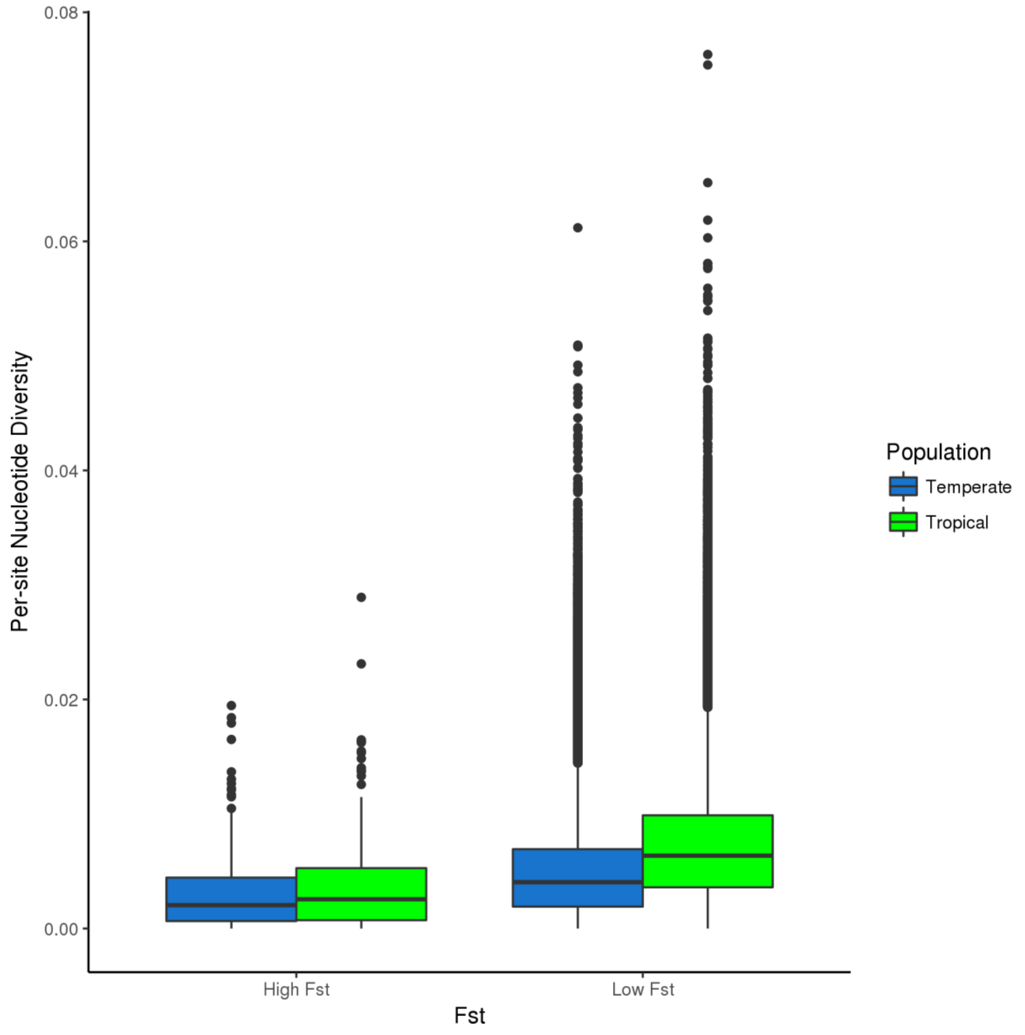

<u>Supplementary Figure 5: Nucleotide diversity distributions at high and low Fst loci</u>

Distributions of nucleotide diversity within high and low Fst windows within 30 temperate and 30 tropical inbred lines.

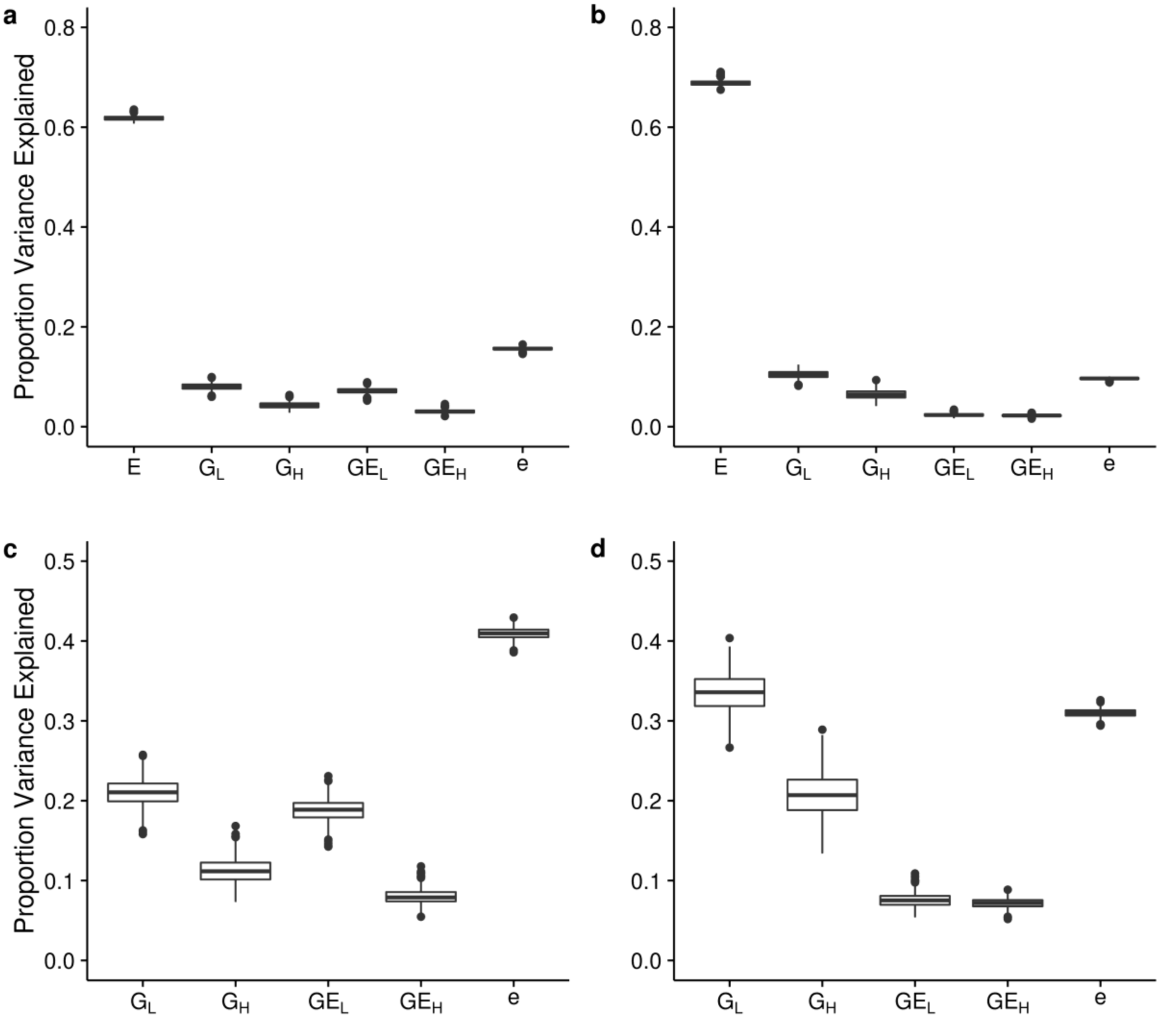

Supplementary Figure 6: Distributions of variance components showing genetic effects of high and low Fst SNPs

Proportions of overall variance attributed to model terms from 1,000 iterations of model fitting with 1,248 high Fst SNPs and subsamples of 1,248 low Fst SNPs taken from the full set of 263,243 low Fst SNPs. Phenotypes were grain yield (a) and plant height (b). Proportions of non-environmental variance (i.e., proportions were calculated excluding environmental variance estimate) for grain yield (c) and plant height (d). Variance components are E: environment, G_L: genotype at low Fst SNPs; G_H: genotype at high Fst SNPs; GE_L: Genotype by environment of low Fst SNPs, GE_H: Genotype by environment of high Fst SNPs, e: residual.

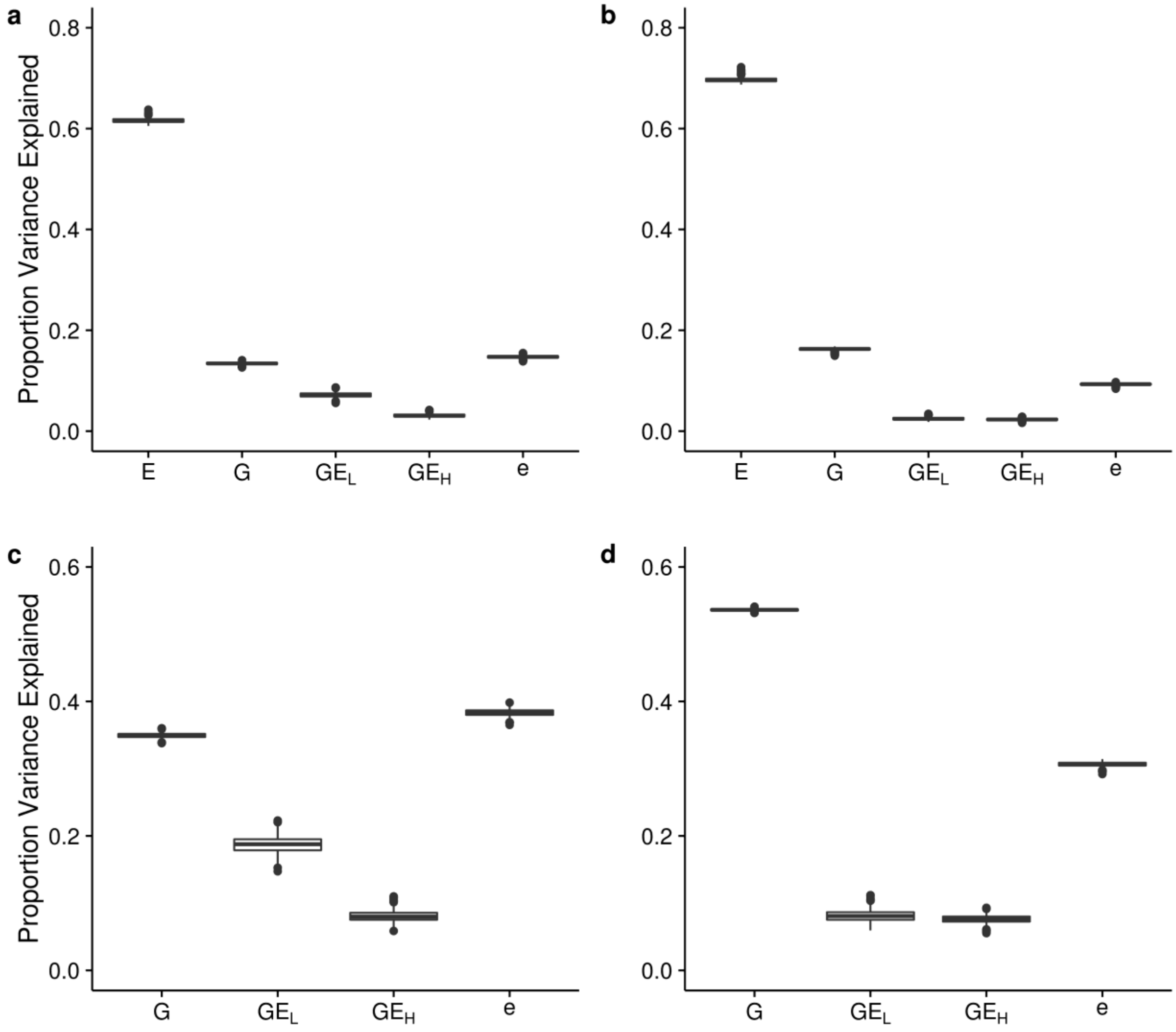

<u>Supplementary Figure 7: Distributions of variance components with a single genetic effect term</u>

Proportions of overall variance attributed to model terms from 1,000 iterations of model fitting with 1,248 high Fst SNPs and subsamples of 1,248 low Fst SNPs taken from the full set of 263,243 low Fst SNPs. Phenotypes were grain yield (a) and plant height (b). Proportions of non-environmental variance (i.e., proportions were calculated excluding environmental variance estimate) for grain yield (c) and plant height (d). Variance components are E: environment, G: genotype, GE_L: Genotype by environment of low Fst SNPs, GE_H: Genotype by environment of high Fst SNPs, e: residual.

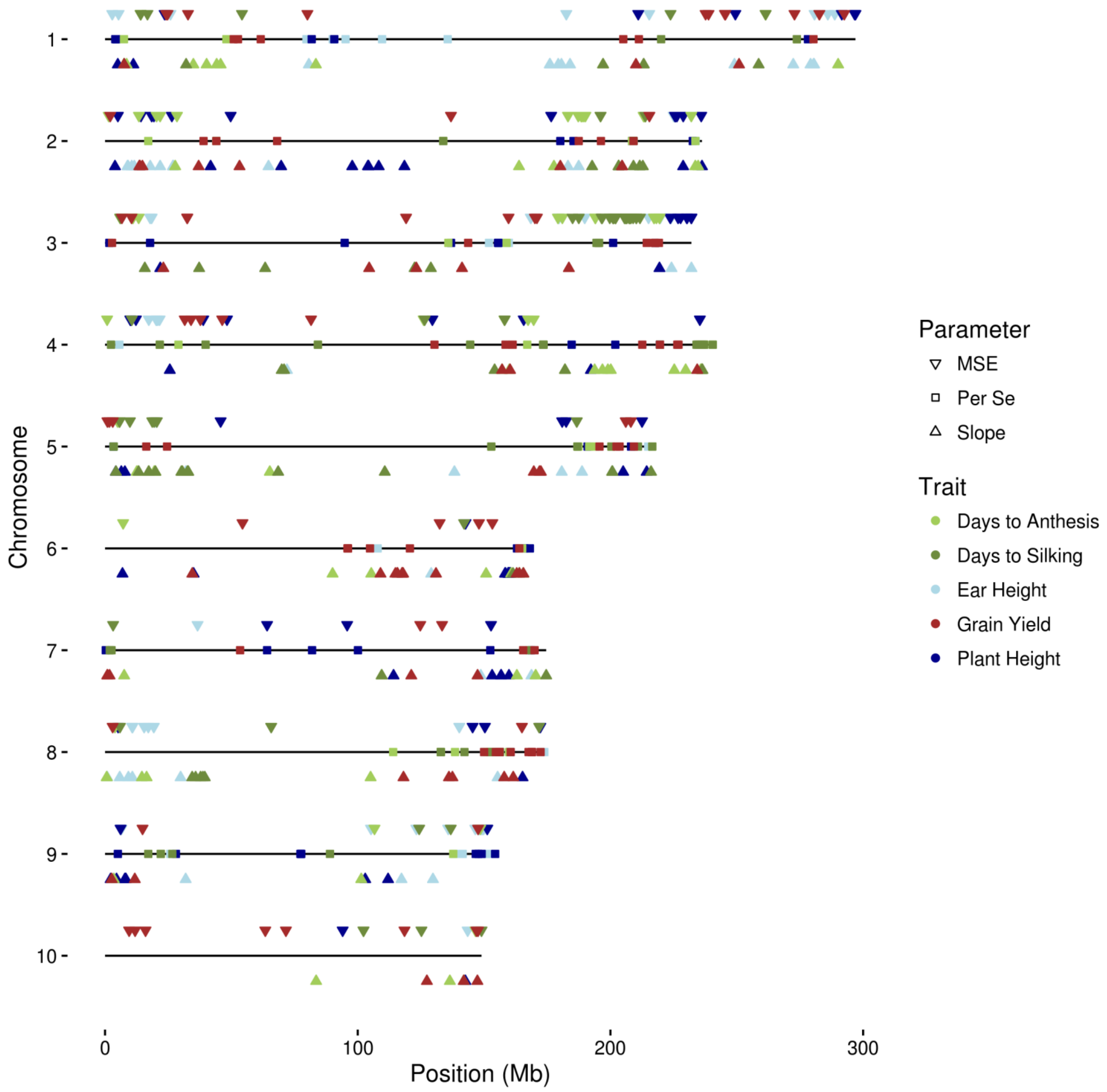

Supplementary Figure 8: Location of slope-, MSE-, and trait-per-se-associated SNPs

Physical locations of 250 slope-associated SNPs, 250 MSE-associated SNPs, and 250 trait-per-se-associated SNPs. Each set of 250 SNPs represents the pooled top 50 SNPs from mapping the relevant parameter for 5 agronomic and phenological traits.

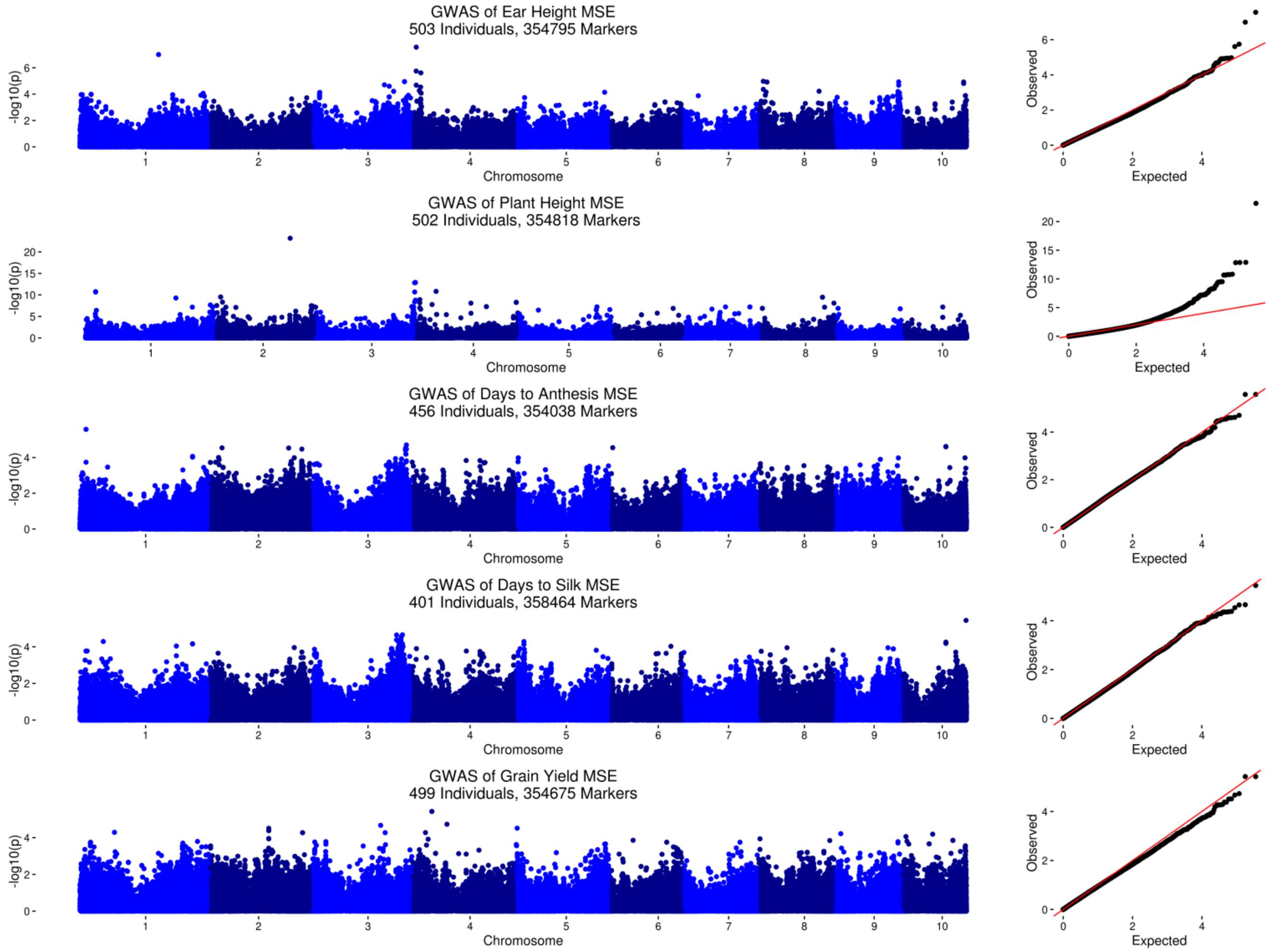

<u>Supplementary Figure 9: GWAS Manhattan plots for MSE</u>

Manhattan plots from performing GWAS using MSE of a Finlay-Wilkinson regression as the response variable.

QQ Plots show the fit of each GWAS analysis.

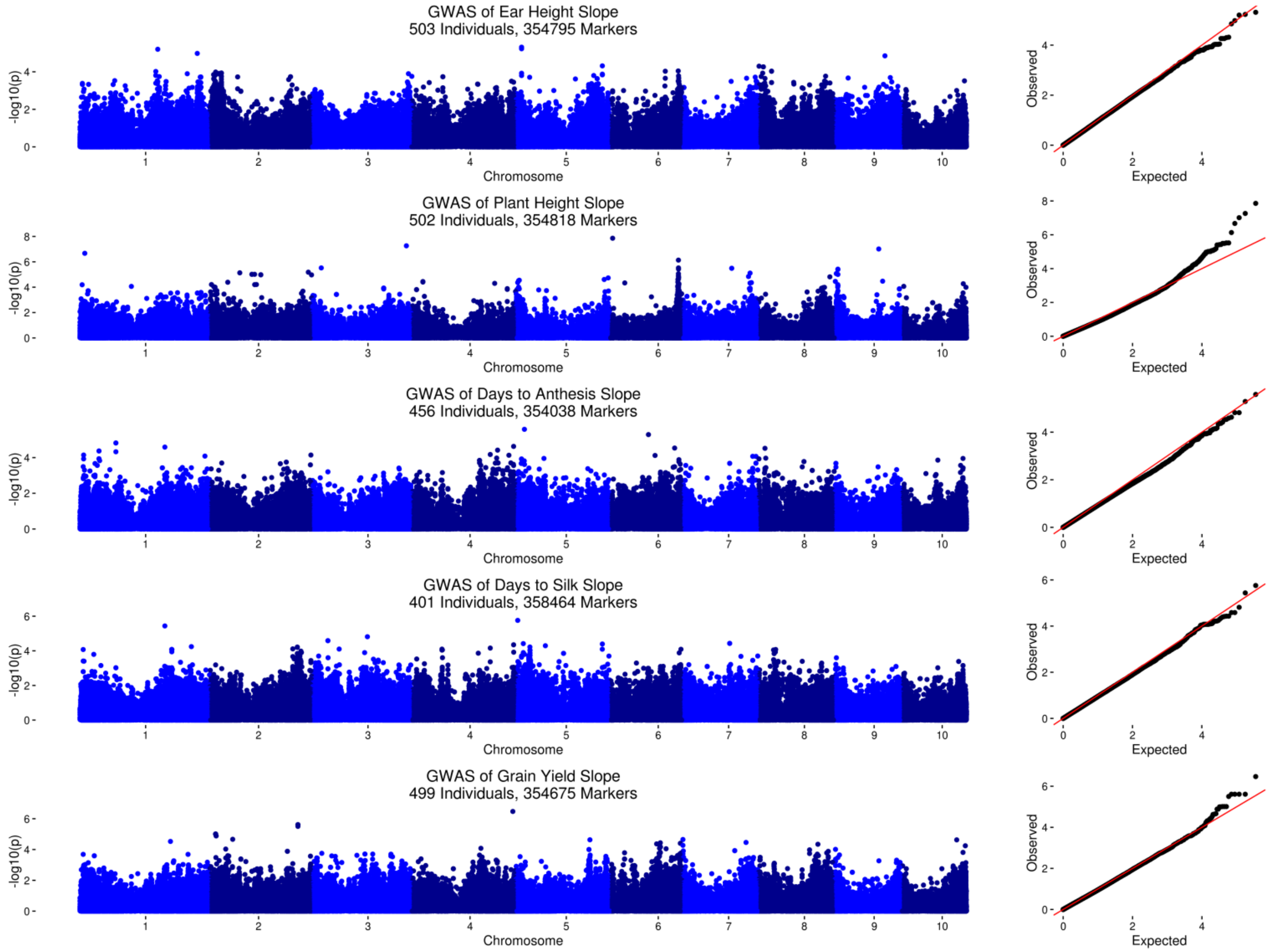

<u>Supplementary Figure 10: GWAS Manhattan plots for slope</u>

Manhattan plots from performing GWAS using slope of a Finlay-Wilkinson regression as the response variable.

QQ Plots show the fit of each GWAS analysis.

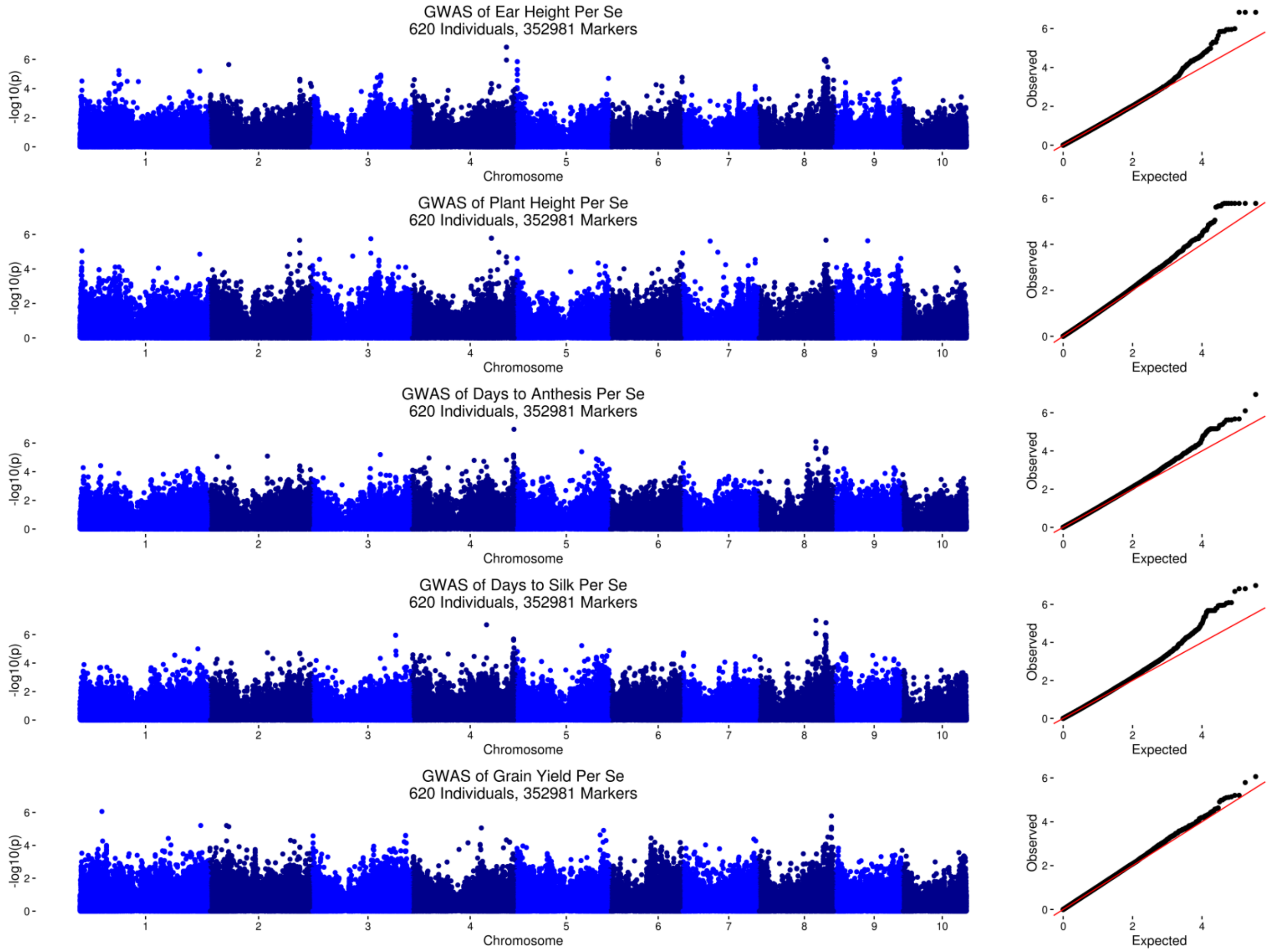

<u>Supplementary Figure 11: GWAS Manhattan plots for traits per se</u>

Manhattan plots from performing GWAS using traits per se as the response variable. QQ Plots show the fit of each GWAS analysis.

Supplementary Table 1: Set formations

Set	Pool	Tester
0		Local checks
1		Common hybrids
2	1	PB80
3	2	PB80
4	3	PB80
5	4,6,7	PB80
6	8	PB80
7	1	LH195
8	2	LH195
9	3	LH195
10	4,6,7	LH195
11	8	LH195
13	1	CG102
14	2	CG102
15	3	CG102
16	4	CG102
17	6,7	CG102
18	8	CG102
19	1	LH198
20	2	LH198
21	3	LH198
22	4	LH198
23	6,7	LH198
24	8	LH198
25	1	LH185
26	2	LH185
27	3	LH185
28	4,5,7	LH185
29	8	LH185

Table of hybrid “sets”, each of which consisted of one or more pools (determined by genetic background) used as females and crossed by the listed male tester.

Supplementary Table 2: Inbreds used to estimate Fst

**Temperate**	**Tropical**
ZEAxppRBSDIAAPEI-4	CML69
B73	KI3
ZEAxppRBHDIAAPEI-1	CML103
ZEAxppRBFDIAAPEI-3	CML52
CAU478	CML333
ZEAxujRALDIBAPE	MAIdgiRABDIAAPEI-2
ye8112	Tzi8
CAUZHENG58	MAIdgiRAXDIAAPEI-6
ZEAxppRCPDIAAPEI-10	CML277
ZEAxppRBRDIAAPEI-3	MAIdgiRCKDIAAPEI-9
W64A	Ki11
Mo17	MAIdgiRAYDIAAPEI-7
ZEAxujRAHDIBAPE	MAIdgiRASDIAAPEI-2
ZEAxujRAPDIAAPE	MAIdgiRAPDIAAPEI-12
ZEAxppRBCDIAAPEI-2	MAIdgiRAWDIAAPEI-5
La2-4	Tx601
zheng32	MAIdgiRCCDIAAPEI-10
D340	MAIdgiRAMDIAAPEI-10
LH74	MAIdgiRAVDIAAPEI-4
T24	MAIdgiRARDIAAPEI-1
DF24	MAIdgiRAKDIAAPEI-9
GY3	NC350
624	MAIdgiRAGDIAAPEI-5
DF20	MAIdgiRACDIAAPEI-3
PHW17	MAIdgiRAADIAAPEI-1
BC4B	MAIdgiRAIDIAAPEI-6
ZEAxppRBHDIAAPEI-5	ZEAxppRBYDIAAPEI-2
H114	NC358
OQ603	MAIdgiRAEDIAAPEI-4
ys06	ZEAxppRCJDIAAPEI-7

A set of 30 temperate inbred lines and 30 tropical inbred lines that were chosen and used to compute Fst.

Supplementary Note 1: Weather data

WatchDogTM Model 2700 (Spectrum Technologies Inc., East-Plainfield, Illinois, 60585, USA) weather stations were placed in the field at each location to measure air temperature (°C), dewpoint (°C), relative humidity (%), rainfall (mm), wind speed (meters * second^{-1}), wind direction (degrees), wind gusts (meters * second^{-1}; largest speed in a 30 minute period) and solar radiation (Watts * meter $^{-2}$) at 30-minute intervals throughout the growing season. Because station placement was left to the discretion of collaborators, observations are considered microscale and are only representative of the field, especially in the case of wind direction. Weather station observations were compared to nearby National Weather Service (NWS) Automated Surface Observing Systems (ASOS) data to verify calibration and identify erroneous data points. Erroneous data points were replaced by a blank. Cleaned and calibrated weather data can be found at doi:10.7946/P2V888. The calibrated dataset includes observations from the NWS ASOS as well as a “calibrated” column for most elements. In cases where a weather element did not require calibration the data was simply copied from the regular data column.
