# Supplementary material for: The effect of artificial selection on phenotypic plasticity in maize
Source: Nat Commun. 2017 Nov 7;8:1348. doi: 10.1038/s41467-017-01450-2 (PMC5677005; doi:10.1038/s41467-017-01450-2)
Supplement: Supplementary file 3 — Descriptions of Additional Supplementary Files [file 41467_2017_1450_MOESM3_ESM.pdf]

Description of Additional Supplementary Files

File Name: Supplementary Dataset 1
Description: Genotyping-by-sequencing inbred sample identifiers and line names

File Name: Supplementary Software 1
Description: Scripts used for estimating high- and low-Fst SNP by environment interactions
